# Optimistic narrative future visions: a communication tool for promoting sustainable (plastic) behavior

**DOI:** 10.3389/fpsyg.2023.1252895

**Published:** 2023-09-18

**Authors:** Nicolas E. Neef, Selina Fußwinkel, Claudine Roos, Lilli Frank, Kapandu Shihepo, Isabell Richter

**Affiliations:** ^1^Department of Sustainable Development and Change, Institute of Education, Work, and Society, University of Hohenheim, Stuttgart, Germany; ^2^Department of Psychology, Norwegian University of Science and Technology, Trondheim, Norway; ^3^School for Geo- and Spatial Sciences, North-West University, Potchefstroom, South Africa; ^4^Unit for Environmental Sciences and Management, North-West University, Potchefstroom, South Africa

**Keywords:** plastic pollution, plastic bags, narrative future vision, video, South Africa, sustainable behavior, environmental communication

## Abstract

Plastic pollution is a pressing global issue, necessitating a focus on consumer behavior to curb this problem at its source. To effectively promote sustainable practices, communication strategies that employ future visions have gained attention. This study examines the effects of a narrative video intervention depicting an optimistic future vision concerning single-use plastic bag consumption in South Africa, compared to a representation of the prevailing *status quo*. Using a preregistered within-subject design, we assess the psychological and emotional responses to two scenarios of which one is illustrating adaptive behaviors toward reduced plastic bag usage, and the other showcasing current consumption patterns. Parametric analyses revealed a shift in emotional states, characterized by a greater experience of positive emotions and a reduced experience of negative emotions following the exposure to the optimistic future scenario video, as compared to the *status quo* video. Moreover, engagement with the optimistic future scenario was associated with higher levels of perceived behavioral control and behavioral intentions. No significant changes were found regarding sense of responsibility. These findings point to the potential of optimistic future visions to influence individuals at psychological and emotional levels. This renders optimistic future vision communication as an effective tool for sustainable behavior change, particularly in relation to the sustainable use of plastic shopping bags.

## 1. Introduction

Plastic pollution is a significant and pervasive issue with detrimental effects on ecosystems, socio-economic systems, and human health (Cavaliere et al., 2020; Kumar et al., 2021; MacLeod et al., 2021). Globally an estimated 29 million metric tons of plastic waste infiltrated both aquatic and terrestrial ecosystems in the year 2016 (Lau et al., 2020). Due to a combination of factors, including a high incidence of mismanaged plastic waste (Pucino et al., 2020), an above-average annual per capita plastic waste generation (IUCN-EA-Quantis, 2020), and significant quantities of plastic entering the water annually via the long coastline (Pucino et al., 2020), South Africa is considered a particularly problematic area in terms of plastic pollution, amplified by a subpar implementation of plastic pollution strategies and regulations (Stafford et al., 2022; Venter, 2022). Currently, South Africa produces an average of 41 kilograms of plastic waste per person on an annual basis, surpassing the global average of 29 kilograms. Furthermore, it holds the distinction of having the highest rate of mishandled plastic waste among African nations (Nyathi and Togo, 2020; Meijer et al., 2021). Although South Africa has developed regulations to phase-out plastic bags by imposing a levy, the consumption of single-use plastic bags remains problematic. By plastic bags we mean those shopping bags that are made for single use (also referred to as “plastic carrier bags” or “plastic grocery/shopping bags” in the South African context). The country’s annual consumption of 8 billion single-use plastic bags (around 0.41 per capita per day; Dikgang et al., 2012; O’Brien and Thondhlana, 2019), necessitates guiding individual consumer behavior toward sustainable plastic behavior (Heidbreder et al., 2023). As we highlight in the subsequent section, various psychological and structural strategies have been previously examined to address the excessive proliferation of plastic bags in South Africa and other regions across the globe (Luo and Zhao, 2023). Nevertheless, a valuable instrument which has been understudied is the utilization of narrative future visions as an effective means of environmental communication (Neef et al., 2023a). We define narrative future vision as a story-based depiction of optimistic potential future scenarios (Richter et al., 2023a). Further, environmental communication itself plays a crucial role in transforming people’s plastic related behavior since it has the potential to affect individuals’ emotions, intentions, and ultimately, behavior (Klöckner, 2015b). Against this backdrop, the primary objective of this study is to address the following research question.

RQ: *Can optimistic narrative future vision scenarios be effectively employed to influence the psychological factors that determine plastic behavior, thereby serving as a potential tool for shaping and influencing plastic-related behaviors?*

In order to address our research question, we will expound upon the current status of structural and psychological interventions pertaining to plastic bags, specifically focusing on the South African context. Our approach employs an optimistic narrative future vision scenario, conveyed through a video-based medium. Consequently, we will scrutinize the ongoing research concerning video-based communication and its effectiveness in conveying optimistic future vision scenarios, particularly in relation to the recognized psychological determinants that influence plastic behavior. Subsequently we outline the procedural and methodological aspects of our study, followed by a the presentation of the analysis outcomes, leading into a comprehensive discussion of the findings. Finally, we will deduce research and practical implications from the outcomes and provide a glimpse into potential future research endeavors. If the effectiveness of video-based narrative future visions in significantly reshaping the behavioral determinants underlying plastic behavior is established, this approach could emerge as a novel, easy to distribute, accessible and reusable tool within the arsenal of both researchers and practitioners (Pressgrove et al., 2021).

## 2. Background

### 2.1. Literature review

#### 2.1.1. Structural interventions to regulate plastic bag consumption

In a bid to address the persisting inundation of plastic, the South African government has implemented a legislative levy on plastic bags and is actively pursuing a directive to permit only the usage of plastic bags composed entirely of 100% recycled material by the year 2027 (Department of Environment Forestry and Fisheries Republic of South Africa, 2021). Nevertheless, evidence has indicated that the early success of the levy, characterized by a reduction in bag consumption, was of fleeting nature, as the initial levels were swiftly regained (Dikgang et al., 2012). This sets South Africa apart from other countries, such as the United States (Wagner, 2017) or the United Kingdom (Thomas et al., 2019), where the implementation of levy systems yielded sustained success. Furthermore, although recycled plastic bags represent a substantial advancement over their virgin plastic counterparts in terms of environmental impact (Stafford et al., 2022), regulations pertaining to the composition of plastic bags may not necessarily exert a significant influence on consumption patterns (Hasson et al., 2007; Adeyanju et al., 2021). Others suggest the replacement of conventional plastic bags with biodegradable alternatives (Hariani and Al Hakim, 2022), offering advantages such as a reduced duration of presence in the environment. Nevertheless, it is crucial to recognize that misunderstandings regarding bio-plastic bags could potentially result in rebound effects (Ansink et al., 2022), where individuals mistakenly presume that bio-plastic bags are much less detrimental to the environment, leading to increased consumption and negligence in recycling efforts and hence an unchecked entry into ecosystems. It can thus be inferred, that in contrast to certain global regions where price mechanisms and the availability of substitutes are foreseeable (Raab et al., 2023), South African consumers demonstrate a comparatively reduced sensitivity to pricing, and the adoption of alternatives might yield limited ecological advantages. Another structural transformation frequently suggested involves the outright prohibition of plastic bags (Wagner, 2017; Raab et al., 2023). Nevertheless, it is imperative to bear in mind that supermarkets derive substantial profits from the sale of bags–one estimate is that major supermarkets in Australia could earn more than $1 million a year (Mortimer, 2017) and that managing the substantial presence of informal supermarkets in South Africa (Ligthelm, 2013) presents a formidable challenge. It is thus questionable if a ban would be a popular legislative intervention amongst business, while even in the event of attaining success, the fundamental issue, i.e., the excessive consumption of bags, albeit composed of different materials, remains an underlying concern. This underscores the necessity for a shift in mindset among South African consumers toward less consumption, as acknowledges by Raab et al. (2023) and exaggerated by the fact that South African consumers acknowledge plastic bags as a concern yet continue to use them due to the convenience they offer (O’Brien and Thondhlana, 2019; see also Khanal, 2022; Misgana and Tucho, 2022). This transformation is crucial to curbing the initial consumption of plastic, rather than solely relying on regulatory alterations, although changing situational factors is often rightfully applied and can be very effective (Heidbreder et al., 2019). Hence, the pivotal emphasis of this study is directed toward fostering a culture of plastic bag reuse, irrespective of their composition. When bags are reused, it is typically linked with the least environmental impact (Gómez and Escobar, 2022). For the present study the focus lies on the reuse of plastic bags, since they are the most commonly found carrier bags in the formal economy sector in South Africa (Russo et al., 2020).

#### 2.1.2. Psychological interventions to regulate plastic bag consumption

A multitude of studies performing behavioral interventions have been presented, focusing on sustainable plastic bag behavior. In an extensive study, Luo and Zhao (2023) meticulously investigated the efficacy of 12 distinct behavioral interventions aimed at diminishing hypothetical consumption of plastic produce bags, which are often distributed at vegetable counters in supermarkets. Their findings revealed that indirect incentives or penalties (achieved through donations to environmental organizations), highlighting the positive outcomes of abstaining from vegetable bags, utilizing normative messages, emphasizing the choice of refraining from vegetable bags, and invoking reminders or visualizations of the adverse consequences of using such bags exhibited the most substantial effects. Heidbreder et al. (2020) examined a campaign encouraging a plastic free month. Their findings revealed that individuals who received information about this campaign exhibited reduced plastic consumption compared to those who were not provided with such information. Regarding the context of South Africa, Abiola et al. (2023) tested both monetary and non-monetary interventions. Notably, within their investigation, the non-monetary intervention, encompassing a plastic-free monthly campaign, similar to Heidbreder et al. (2020), demonstrated greater effectiveness compared to the two tested monetary interventions, namely, a one-time distribution of reusable plastic bags and a subsidy for reusable bags. However, to enrich the repertoire, rather than duplicating the aforementioned studies within this investigation, we intend to adopt a distinct approach, focusing on environmental communication through narrative future visioning.

### 2.2. Conceptional framework

#### 2.2.1. Videos as a communication tool

In recent years, there has been a growing recognition of the need for enhanced communication strategies to effectively utilize pro-environmental communication interventions and maximize their persuasive impact (Klöckner, 2015b; Xexakis and Trutnevyte, 2021). In this vein, previous research has explored the effects of future vision communication (Richter et al., 2023a). These visions are presented through various media formats, such as infographics (Richter et al., 2021), drawings (Löfström and Klöckner, 2019), animated pictures (De Munnik and Lenzholzer, 2020), and photorealistic visualization (Tress and Tress, 2003), as well as interactive formats like virtual reality or board- and video games (Angel et al., 2015; Vervoort et al., 2015; Blythe et al., 2021). The incorporation of visual media formats within environmental communication stands as a central recommendation (Klöckner, 2015a), aligning with the innate human inclination toward visual content. Visuals possess the capability to captivate our attention, evoke potent emotions surpassing textual information, offer swift cognitive processing, and present a cost-effective implementation (Löfström et al., 2021). In fact, certain scholars contend that visualizations stimulate our motivation by activating pertinent objectives (Zlatev et al., 2010). Indeed, visuals possess persuasive power in shaping values, norms, and worldviews, and are successful in promoting environmentally responsible practices (Perrin, 2011; Blythe et al., 2021). However, studies specifically focusing on future vision communication presented in the form of videos are relatively sparse. The effectiveness of video-based interventions in influencing behavior was initially demonstrated in research on health-related behaviors (Tuong et al., 2014). As such, videos have gained popularity as a communication tool in the field of environmental communication (Warner et al., 2018). Videos offer a compelling means of presenting information through both auditory and visual elements, facilitating quick comprehension and engagement (Geise and Baden, 2015). Considering the rising prevalence of videos in everyday life, particularly through social media platforms, they have become increasingly significant as communication tools and have emerged as a standard means through which individuals access information and entertainment (Trimble, 2015; Zhang et al., 2022). Yet, there is limited knowledge regarding the most effective methods specifically for video-based communication to promote sustainable behaviors (Moskell and Turner, 2022; Yang et al., 2022). Nevertheless, videos employing storytelling techniques offer a promising yet underexplored approach for communicating pro-environmental behavior (Neef et al., 2023a).

#### 2.2.2. Optimistic narrative future visions

Evidence suggests that narratives are more easily understood and appealing to audiences than tables, infographics, or diagrams and can capture attention and evoke emotions more effectively compared to standard scientific communications (Green, 2006; Dahlstrom, 2014). A narrative can be defined as a “cohesive and coherent story with an identifiable beginning, middle, and end that provides information about scene, characters, and conflict; raises unanswered questions or unresolved conflict; and provides resolution” (Hinyard and Kreuter, 2007). Narratives possess persuasive power by captivating viewers and immersing them in the story’s world, leading to identification with characters (Slater and Rouner, 2002). Narratives are thus especially successful when they feature one or more protagonists faced with analogous challenges within comparable environments as the audience. This similarity between the protagonist and the visionaries or audience cultivates a profound sense of affinity and connectivity (Richter et al., 2021), enabling a sense of identification (Pahl and Bauer, 2013). A successful narrative transportation can potentially overshadow existing cognitive frameworks, knowledge, experiences, and opinions (Morris et al., 2019), potentially reducing resistance to the message and facilitating the adoption of intentions and behaviors aligned with the story’s protagonist (Green and Brock, 2000; Niederdeppe et al., 2011). Previous research focused on promoting pro-environmental behavior change has provided evidence that narratives contribute to increased levels of pro-environmental knowledge and attitudes (Yang et al., 2022), stronger behavioral intentions (Nakano and Hondo, 2023), and ultimately, the adoption of more sustainable behaviors (Morris et al., 2019). In addition to these general positive effects of narratives, there is specific research supporting the favorable outcomes of the inclusion of optimistic future visions within narrative structures (Richter et al., 2023a). These outcomes encompass an improved understanding of future consequences and perceived behavioral control, heightened empathy toward environmental issues, and ultimately, enhanced motivation and intention to engage in pro-environmental behaviors (De Munnik and Lenzholzer, 2020; Blythe et al., 2021; Richter et al., 2021). Particularly, optimistic future visions are regarded as critical for facilitating sustainable transformations as they serve as sources of aspiration, motivation, and foundations for initiating tangible actions (McPhearson et al., 2016; Richter et al., 2021). Such visions elicit positive emotions like hope and empowerment, which exert significant influence on pro-environmental intentions and behaviors (Khalil et al., 2022; Stadlthanner et al., 2022). Against this background, the intervention employed in this study combines the advantages offered by video-based communication, the persuasive influence of narratives, and the promising potential of optimistic future visions to actively engage people with the presented issue to promote more sustainable plastic behavior.

### 2.3. Theoretical framework

Within this section, we expound upon the variables that our project aspires to impact, considering the context of the video-based narrative we employ and formulate our hypothesis on the way. One factor that is frequently and consistently associated with intentions to engage in sustainable plastic behavior is perceived behavioral control (PBC; e.g., Nabila et al., 2020; Van et al., 2021), a component of the theory of planned behavior (TPB; Ajzen, 1991). It is defined as a person’s subjective perceived difficulty to perform a behavior. According to the TPB, PBC directly influences sustainable behaviors and also indirectly influences them through intentions (T’ing et al., 2020; Fang et al., 2021). For example, a study focusing specifically on plastic pollution behavior found positive effects of PBC on reduced plastic usage (Hasan et al., 2015). The positive relationship between intentions and actual behavior has also been supported by multiple studies. Strydom (2018) and Fang et al. (2021) revealed the positive influence of recycling intentions on recycling behavior, while Arı and Yilmaz (2017) demonstrated the intention to use fewer disposable bags corresponding to a reduction in actual bag usage. One approach to enhance PBC and consequently strengthen intentions and behaviors is by presenting individuals with alternative courses of action and fostering self-efficacy (Ajzen, 2002) and thus increase the external and internal scope for action. Concerning narrative future vision scenarios, both aspects can indeed contribute to success. On one hand, the narrative can effectively depict alternative actions through protagonists, while simultaneously bolstering self-efficacy by showcasing the potential for successfully evading plastic through these alternative choices. The remaining two variables of the TPB, namely, attitude and subjective norms, also exhibit correlations with plastic behavior (Nabila et al., 2020; Van et al., 2021). Nonetheless, it is important to underscore that attitudes toward single-use plastic bags are frequently already very negative across various regions (Khanal, 2022), including South Africa (Richter et al., unpublished). Indeed, data gathered through the TPB frequently reveal significant floor or ceiling effects, leading to insufficient variability for meaningful statistical analyses–a phenomenon acknowledged by La Barbera and Ajzen (2020). Given the expectation of a prevailing floor effect concerning attitudes toward plastic bags in this instance, we opted to exclude this variable from our study. On the other hand, subjective norms hold promise and can serve as a potent catalyst for change if modified (Neef et al., 2023a). Yet, it is essential to acknowledge that altering these norms may entail a protracted and intricate process, which might not be immediately feasible within the scope of this study. We thus decided to also omit this variable focus on PBC. Indeed, research has demonstrated that PBC offers a prime foundation for altering intentions and effecting behavioral changes within the context of the TPB concerning plastic-related actions (Sun et al., 2017). Thus, PBC emerges as the variable that we anticipate being most amenable to change and to wield the most pronounced influence, leading to the following hypothesis.

*H1: Viewing an optimistic narrative future vision scenario increases participants perceived behavioral control.*

Another variable linked to plastic avoidance is the cultivation of a sense of responsibility toward plastic pollution. It is imperative for individuals to harbor a genuine sense of responsibility to prompt a reevaluation of their behavior (Klöckner, 2013). Therefore, an elevated sense of responsibility serves as a reliable predictor of alterations in intentions and ultimately sustainable behavior (Han, 2015). It has been observed that as individuals perceive themselves as more accountable for environmental issues resulting from their actions, their likelihood of engaging in pro-environmental behavior increases (Okumah et al., 2020). Specifically, in the context of reducing plastic waste, research has indicated that when individuals believe that corporations (instead of individuals) bear the responsibility for reducing plastic waste, they are less inclined to engage in recycling (Thanh et al., 2012) or use reusable bags (Romero et al., 2018). Furthermore, the sensation of powerlessness often stands in contrast to one’s personal sense of responsibility. Fortunately, within a narrative scenario, the protagonist’s positive actions can alleviate this feeling and subtly underscore the individual’s responsibility (Richter et al., 2023a). This leads us to the following hypothesis.

*H2: Viewing an optimistic narrative future vision scenario increases participants’ sense of responsibility.*

Both PBC and a sense of responsibility are linked to intentions (Klöckner, 2013). Consequently, bolstering these two variables should result in a corresponding enhancement of intentions. Moreover, when displayed in narrative future scenarios the intentions of protagonists are readily apparent and relatable to the audience and thus could potentially elevate the personal intentions of the viewers (Neef et al., 2023a). Against this background we formulate the following hypothesis.

*H3: Viewing an optimistic narrative future vision scenario increases participants’ behavioral intention (toward a more sustainable consumption of plastic bags).*

**FIGURE 1:**
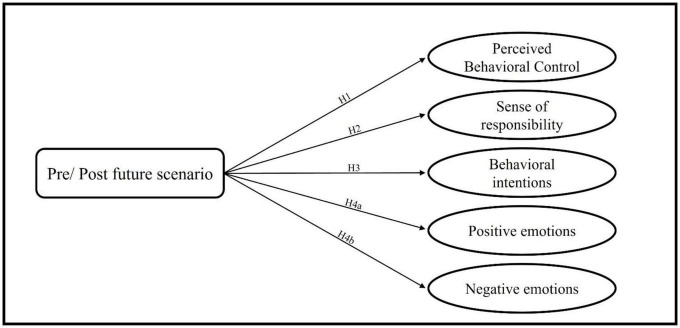
A summary of the hypotheses.

A common explanation for the found disparity between acknowledging environmental issues and translating that recognition into action may potentially stem from a deficiency in emotional engagement (Roeser, 2012; Löfström et al., 2021). Emotions significantly impact the decisions we make on a daily basis, thus shaping our behavior (Rezvani et al., 2017). Specifically, in the context of sustainable behavior research, positive emotions, such as hope or happiness, have been found to increase behavioral intentions (Onwezen, 2015) and encourage pro-environmental behavior (Ojala, 2012). The relation between negative emotions and pro-environmental behavior is a more multifaceted one. Research has demonstrated that certain negative emotions, such as guilt, shame, or indignation, can actually promote pro-environmental behavior (Brosch and Steg, 2021; Ibanez and Roussel, 2021). However, negative emotions like fear, anxiety, and distress tend to hinder such behavior (Tapia-Fonllem et al., 2010). In our study, we focused on the latter aspect, that is focusing on those negative emotions inhibiting pro-environmental action. Against this background we formulate our final hypotheses a summary of all hypotheses can be found in Figure 1.

*H4a: Viewing an optimistic narrative future vision scenario increases participants’ positive emotions.*

*H4b: Viewing an optimistic narrative future vision scenario decreases participants’ negative emotions.*

## 3. Materials and methods

### 3.1. Procedure and data collection

Our preregistered study^1^ was conducted as an online survey using the SoSciSurvey platform (Leiner, 2019). The survey was administered in English and took approximately 15 min to complete. Socio-demographic data were collected at the beginning of the study. Designed as a Within-Subject design, all participants were first exposed to a *status quo* scenario (henceforth *status quo*), depicting a realistic shopping process in a typical South African supermarket. Subsequently, participants answered the items measuring behavioral intentions, sense of responsibility, perceived behavioral control, and positive and negative emotions. Afterward, an optimistic narrative *future vision* video (henceforth Future Vision) was presented, and participants re-evaluated the same items. To ensure that participants attentively followed the videos and did not encounter any viewing problems, they were asked a question about the video’s content and whether they experienced any technical difficulties after each video.

### 3.2. Sample

To determine the required sample size for a dependent sample *t*-test with a mean effect of Cohen’s *d* = 0.2, a power of 0.95, and an alpha error rate of 0.05, we performed an *a priori* power analysis. The determined sample size was 272 subjects. Due to expected exclusions, the collection of 300 valid data sets was set as a target. Participants were recruited via a panel selection process (81.25%) and emails (18.75%). The inclusion criteria for this study involved attaining an age of 18 years or older and being a resident of South Africa. It was attempted to achieve a representative sample of the South African population, in terms of age and gender of the participants. We adhered to our previously established data exclusion policy (see text footnote 1), including exclusion for the following reasons: Failure to pass the attention test (incorrect answer to control questions), reporting technical problems, participants’ completion time was too short, indicating low data quality, or more than 20% of responses were missing. Overall, 384 datasets were collected, of which 42 had to be excluded due to the mentioned criteria. As a result, 342 valid datasets were available for data analysis. Gender and age distribution of the final sample are provided in Table 1. With an age median of 28 years, the South African population is relatively young, while the sex ratio of 0.98 indicates a slight predominance of females over males within the population (Central Intelligence Agency, 2023). In our sample, this trend is similarly reflected, albeit to a more pronounced extent, due to the slight over-representation of female participants.

**TABLE 1:** Sample description.

Age categories	Gender distribution	*n*	%
18–29 years	Total	109	31.87%
	Male	40	11.70%
	Female	67	19.59%
	Diverse	1	0.29%
	Non-binary	0	0%
	Not specified	1	0.29%
30–39 years	Total	108	31.58%
	male	40	11.70%
	Female	67	19.59%
	Diverse	0	0%
	Non-binary	1	0.29%
	Not specified	0	0%
40–49 years	Total	58	16.96%
	Male	26	7.60%
	Female	32	9.36%
	Diverse	0	0%
	Non-binary	0	0%
	Not specified	0	0%
>50 years	Total	67	19.59%
	Male	43	12.57%
	Female	24	7.02%
	Diverse	0	0%
	Non-binary	0	0%
	Not specified	0	0%

### 3.3. Development and content of the video material

The animated videos employed in this study were previously published as part of the research conducted by Neef et al. (2023a) and were collaboratively developed in conjunction with diverse South African stakeholders. In the aforementioned study the rationale for their formation, specifically collaborative development method, is explicated. They depict a shared plotline, but they are deliberately designed to have different outcomes, offering viewers distinct perspectives and narrative directions. Below we describe the plot differences in short. For more comprehensive information on the development and content of the two videos, refer to Neef et al. (2023a). For assessing the videos see the links to the videos in the Supplementary material (p. 6).

#### 3.3.1. *Status quo* video

The video depicting the *status quo* scenario was developed in collaboration with project partners from South Africa, ensuring the authenticity of the protagonists depicted in the video and the portrayed shopping experience for South African consumers. The animated clip follows the journey of Thandi, a woman in her middle years, engaging in a lifelike shopping experience. Thandi makes the choice to head to a supermarket in her car and proceeds to load up her shopping cart with a variety of items, encompassing food, sanitary goods, and bottles of wine. As she reaches the checkout counter, Thandi is presented with a range of bag options, including cotton, paper, and plastic. Opting for plastic bags, a packer begins to pack her chosen items.^2^ After Thandi returns home, she proceeds to unpack her groceries, and subsequently discards the bags by placing them directly into the trash. The video has a duration of approximately one and a half minutes.

#### 3.3.2. Future vision video

**FIGURE 2:**
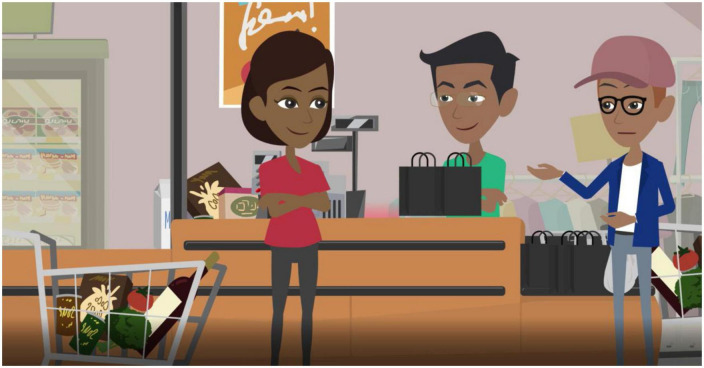
A screenshot of the Future Vision video showing the protagonist **(left)**, the cashier **(middle)**, and another customer **(right)** at the checkout in a grocery store.

As an alternative version of the *status quo* video, an optimistic Future Vision video was developed in 2022 through a joint visioning workshop involving various stakeholders from the public, economy, academia, and environmental conservation sectors (for a comprehensive list of involved stakeholders and respective represented sectors please see Supplementary Table 1). The initial plot remained the same compared to the *status quo* video, however, many key details have been changed to create an optimistic scenario that illustrates pathways to more sustainable usage of plastic bags. Specifically, economic, structural, and behavioral modifications have been added to the video, which were deemed most effective within the South African context by the experts taking part in the workshop. When Thandi leaves the house to go shopping she recalls seeing a sign promoting a special offer (a 5% discount for the initial 1,000 customers who bring their own bags). She thus retrieves shopping bags from her home. As she reaches the till, Thandi is informed that the store has replaced disposable plastic bags with reusable ones as part of their efforts to minimize environmental impact. With her own bags unable to accommodate all her purchases, another customer kindly lends Thandi an extra bag. She opts to use this bag instead of buying more. Thandi’s shopping meets the criteria for the promotional discount, leaving her content as she departs the store, having both reduced her plastic waste and saved money. Upon returning home, Thandi consciously stores the bags for future use instead of discarding them. She also attached a note to the door to remember to bring them on her next shopping trip. Additionally, she ensures there’s always a bag in her car’s trunk. Motivated, Thandi talks about her shopping adventure to her family and friends, ultimately leading them to start a neighborhood initiative encouraging others to reuse bags. The press takes notice of their endeavors, and as a result, Thandi and her fellow campaigners receive media attention. The video has a duration of approximately 4 min, with a screenshot provided in Figure 2.

### 3.4. Measures

The psychological and emotional impacts of the implemented communication intervention were evaluated using the variables of sense of responsibility, perceived behavioral control (PBC), behavioral intentions, as well as positive and negative emotions, including the basic emotions such as worry, anger, hope and empowerment (see Nicholson-Cole, 2005; Sheppard, 2005). Participants provided responses on a 5-point Likert scale, ranging from “strongly disagree” to “strongly agree.” The selection of items has been based on previous research on future scenarios and creative communication (e.g., Richter et al., 2021) and stakeholder consultation during the joint visioning workshops.

Sense of responsibility and PBC were assessed using single items, with the former item being “*This video makes me feel responsible for plastic pollution in my community*” and the latter item being “*This video makes me feel that it is difficult to tackle plastic pollution as an individual.*” Intentions were measured using a 5-item scale, which included items such as “*This video motivates me to reduce my plastic bag consumption.*” These items are derived from Klöckner and Blöbaum (2010) with appropriate modifications made to suit the specific context of the present study. Positive (hope and empowerment) and negative (worry and anger) emotions were assessed using two items each, adapted from the Positive and Negative Affect Schedule (PANAS; Watson et al., 1988). Illustrative items used are “*This video makes me feel worried*” to measure negative emotions and “*This video makes me feel hopeful*” for positive emotions (see Supplementary material for the full questionnaire; pp. 7–14).

### 3.5. Analysis approach

All statistical analyses were conducted using RStudio Team (2020). R-packages used are car (Fox and Weisberg, 2019) and psych (Revelle, 2023). Prior to analysis, the data sets from the email and panel respondents were merged. Descriptive statistics were computed for all study variables, including mean scores and standard deviations. PBC was recoded according to the reversed wording of the item. The five items assessing behavioral intentions and the two items measuring positive and negative emotions were combined into scales by calculating the mean scores. The scale values were only formed if all items of a scale were answered. The intention scale demonstrated high internal consistency at both measurement points, with Cronbach’s α = 0.82 at the first measurement point and α = 0.85 at the second measurement point. The emotions scales showed satisfactory reliability with persons correlation of *r* = 0.67 and *p* < 0.001 for negative emotions before the intervention, *r* = 0.73 and *p* < 0.001 for negative emotions after, *r* = 0.33 and *p* < 0.001 for positive emotions before the intervention and *r* = 0.72, and *p* < 0.001 for positive emotions after. The adequacy of the normal distribution of differences, a prerequisite for performing paired-sample *t*-tests, was assessed by examining histograms (see Supplementary Figure 1). The examination of the histograms revealed satisfactory, albeit imperfect, normal distributions. However, the paired-sample *t*-test is a robust statistical analysis that can withstand violations of normality, and a violation of this assumption is considered unproblematic with a sample size > 30 (Salkind, 2010). Thus, we pursued performing five separate paired-sample *t*-tests, which compared the responses after viewing the *status quo* video with the responses after the Future Vision video. For the sake of robustness, the results of non-parametric Wilcoxon tests are provided in the Supplementary Table 2. In cases where an individual had missing scale values on at least one measurement point, their data for that pair of measurements were excluded from the analysis of the corresponding variable (pairwise case exclusion).

## 4. Results

Table 2 presents means and standard deviations for all study variables after viewing each video and the results of the performed paired-sample *t*-tests.

**TABLE 2:** Means and standard deviations of study variables after viewing each video and results of the dependent sample *t*-tests.

Variable	*N*	*Status quo M*(*D*)	Vision *M*(*D*)	Mean difference	*t*(*df*)	Cohen’s *d*
PBC	341	2.67 (1.33)	3.47 (1.38)	0.80	−9.4 (340)***	0.511
AR	341	4.04 (1.02)	4.09 (1.10)	0.05	−1.1 (340)	0.060
INT	342	4.37 (0.68)	4.58 (0.58)	0.21	−6.9 (341)***	0.373
Pos. Emotion	340	3.50 (0.93)	4.51 (0.74)	1.01	−19.0 (338)***	1.026
Neg. Emotion	340	3.03 (1.16)	1.60 (0.81)	1.43	19.0 (339)***	1.004

*p*-values one-tailed. ***p < 0.001.

The results indicated that after viewing the optimistic Future Vision video, there were significant mean differences for PBC, intentions and the experience of positive and negative emotions. In support of hypotheses H1, H3, H4a, and H4b average scores for PBC, behavioral intentions and positive emotions were significantly higher after the communication intervention, while mean scores for negative emotions decreased significantly. The results revealed no significant changes in the mean scores for sense of responsibility after viewing the *status quo* and the Future Vision video. Consequently, there is no sufficient empirical support for the hypotheses H2. For graphical display of the results see Supplementary Figure 2.

## 5. Discussion

The need to develop effective communication strategies to guide individuals and communities toward more sustainable behaviors has been highlighted in the scientific discourse. In this study, we presented an approach for fostering sustainable consumption of single-use plastic bags in South Africa using an optimistic future vision in video format. The findings from the analyses yielded four principal outcomes. It was demonstrated that engaging with an optimistic future vision scenario leads to an increased perception of behavioral control (H1) and behavioral intentions (H3). While we can assume a medium effect of the intervention for PBC see Cohen’s *d* in Table 2, we only find a small effect for intention change (Cohen, 1988). Additionally, we provided evidence that the exposure to the optimistic narrative future vision resulted in a significant shift in emotional states characterized by a greater experience of positive emotions (H4a) and a reduced experience of negative emotions (H4b) when compared to a *status quo* scenario. Compared to the two proceeding variables where we could only observe small to medium effects, the influence of the future vision video on emotions was large. No significant differences were observed between viewing the *status quo* and Future Vision video regarding the sense of responsibility (H2).

### 5.1. Theoretical implications

Our analyses indicate that the implemented intervention has a positive impact on individuals’ confidence in their ability to adopt sustainable consumption practices of plastic bags. By presenting Thandi’s proactive and environmentally responsible behavior in the optimistic scenario, viewers are not only exposed to an optimistic narrative, but are also provided with several practical examples of how they can contribute to a plastic bag-free lifestyle themselves. That the video is providing valuable behavior alternatives might increase their sense of self-efficacy. As individuals’ behavior is strongly influenced by their perceived ability to perform it (Bandura, 1982, 1991; Ajzen, 1991), the observed effect on PBC (H1) is crucial for potential sustainable behavior change. The significant influence of PBC on the likelihood of performing a certain behavior is not only postulated in established theories, such as the Theory of Planned Behavior (Ajzen, 1991), but has also been demonstrated in prior research connected to reducing plastic usage (Sun et al., 2017; Van et al., 2021). By visualizing practical examples for behavior change, individuals’ confidence in their ability to modify their behavior can be enhanced. The Future Vision intervention may thus help to overcome barriers and facilitate engagement in more sustainable consumption practices, specifically regarding the use of single-use plastic bags.

Further, our study revealed an increase in behavioral intentions toward more sustainable use of plastic bags following the exposure of the Future Vision video. Behavioral intentions are considered a crucial influence on actual behavior (Ajzen, 1991; Klöckner and Blöbaum, 2010), as they indicate the level of effort individuals are willing to exert to perform a behavior, with a general assumption that stronger intentions lead to a higher likelihood of behavior performance (Ajzen, 1991). Is it possible that watching the Future Vision for plastic bag use counteracts the psychological distance people may often feel toward environmental challenges and hence increases intentions to contribute to the positive change. Visualizing a possible positive vision for people’s local environment as described in Sheppard (2012), that does not lie too far ahead see also recommendations for the human time horizon by Tonn et al. (2006), may reduce the distancing effects associated with considering global future scenarios that often lie far in the future (Richter et al., 2023a). The increase in behavioral intentions toward sustainable use of plastic bags signifies individuals’ heightened commitment and motivation to adopt environmentally friendly practices. This finding reinforces the notion that interventions employing optimistic future scenarios can be influential in promoting pro-environmental behavior by shaping individuals’ intentions and ultimately their actions.

For sense of responsibility (H2), no significant changes were observed between the measurement points. As individuals tend to behave in ways consistent with the responsibility they attribute to themselves regarding specific situations and outcomes based on a corresponding sense of obligation (De Groot and Steg, 2009), this factor is highly effective in promoting behavior change (Ariestiningsih et al., 2018). The absence of a significant effect in this regard may potentially limit the effectiveness of the Future Vision intervention. However, within the context of this study, it is important to note that participants’ sense of responsibility was already robust at the initial measurement point following the viewing of the *status quo* video, indicating a ceiling effect (see Table 2). These high levels of responsibility scores make improvement through the Future Vision video less likely due to the participants’ already elevated sense of responsibility prior to the optimistic future vision. Another reason for this could be that the participants did not create the videos themselves [in contrast to Richter et al. (2021) where the participants created their own future scenarios], but were exposed to albeit locally adapted, but pre-produced videos. This could potentially lead to less ownership and responsibility for the topic also discussed in Praet et al. (2023). However, to determine whether the lack of effect is attributable to the initially high levels of responsibility scores or the perceived distance to pre-produced materials, further studies need to be conducted.

Tong et al. (2020) and Khalil et al. (2022) emphasize the positive emotional impact of portraying solution-orientated sustainable behaviors and their positive outcomes. Increased motivation, engagement, and willingness to adopt sustainable behaviors are potential effects (Ojala, 2012). Specifically optimistic future scenarios have been further found to evoke positive emotions in individuals such as hope, empowerment, and a sense of possibility (Krekel et al., 2021; Richter et al., 2023a). Our findings of increased positive emotional states (hope and empowerment) resonate with these findings well. At the same time, our results show that negative feelings of anger and worry decline after viewing the displayed optimistic future scenario compared to the *status quo* video. Specifically, it is plausible that the participants experienced displeasure and anger toward Thandi’s inability to avoid plastic bags in the *status quo* video. This could even be when individuals themselves regularly buy single use plastic bags. Indeed, environmental “misbehavior” of others is frequently explained through factors like ignorance, naivety, or need for convenience, while one’s own unsustainable actions are justified by external factors (Hansmann and Steimer, 2017). Consequently, it is plausible for participants to experience both negative and fewer positive emotions watching the *status quo* video, even if they would behave similarly. However, in the optimistic future rendition of the video, this issue is addressed as Thandi not only takes proactive steps to avoid plastic bags but also initiates a successful campaign against them. It is even possible that the concept of a community coming together to campaign against plastic bags evokes positive emotions and might even instill a sense of collective efficacy in the viewer (Kastner and Matthies, 2023). By showcasing Thandi’s transformation in behavior and the positive outcomes of her choices, the optimistic scenario provides a powerful narrative that can resonate with viewers, motivating them to take similar actions in their own lives.

In conclusion, the observed effects on the measured psychological variables yield positive results in terms of intervention success. In relation to the research question, the employed communication intervention can be considered an effective tool for eliciting psychological and emotional engagement. In the following section, we will discuss the practical and managerial implications of these findings.

### 5.2. Practical and managerial implications

Our research offers significant insights for educators, NGOs, and policymakers. We demonstrate that by collaboratively engaging stakeholders from diverse backgrounds, it is possible to establish innovative solutions for contemporary environmental and societal challenges, as highlighted by Neef et al. (2023a). The findings of the present article suggest that disseminating these innovative ideas as narrative future visions can resonate with a broader audience, potentially inducing wider behavioral change. For optimal impact, it is crucial that these visions reflect local nuances, have a temporal scope of no more than a decade–preferably less–and are shared through accessible means such as short videos, comics, murals, or posters, with Richter et al. (2023a) offering more detailed guidance. The South African Department of Forestry, Fisheries and Environment (DFFE) has set objectives for education and awareness around waste management (including plastic waste) in the country’s National Waste Management Strategy. We believe that the educational nature of the material created through future visions and narratives may be useful toward achieving these educational and awareness creating goals. The judicious choice of social media, tailored to local preferences, can amplify the vision’s reach and influence. Implementing these visions within educational settings can vividly illustrate potential sustainable trajectories and their environmental outcomes. To foster a stronger sense of community stewardship among youth, we do however, recommend to actively involve students in the envisioning process, allowing them to shape their futures, instead of being “only” recipients. Additionally, consistent feedback from the community and stakeholders about the future scenario is invaluable, emphasizing the need for the vision to be fluid, adapting and evolving in tandem with its community. Policymakers need to identify emerging issues, articulate scenarios, develop a shared vision of the desired future, and design robust policies and strategies within complex environments. They may use futures strategic thinking and foresight tools to map different futures and gain a sense of direction to inform policy interventions. Lastly, society’s views on future visions may help policymakers adopt a more proactive approach to solving problems, which are also in line with the attitudes and views of the public. Developing policy which is in line with the views of society may, ultimately, increase buy-in and acceptance by the public, leading to more effective policymaking and implementation.

### 5.3. Limitations and implications for future research

Like all empirical work, this study has several limitations. Firstly, it is essential to consider the level of immersion and transportation into the narrative as crucial factors in determining the persuasive power of the intervention and the recipient’s motivation to adopt recommended actions and attitudes (Green and Brock, 2000; Slater and Rouner, 2002). Therefore, it would have been beneficial to include measurements assessing immersion in our study. A meta-analysis conducted by Braddock and Dillard (2016) on narrative and non-narrative messages emphasized the importance of examining mediating variables such as immersion. Additionally, Zheng et al. (2022) found that the effects of (non-)narrative videos on people’s intention to protect the environment were mediated through immersion experience. By including items measuring these factors, we could have explored whether the lack of effects on behavioral intentions and sense of responsibility could be attributed to a lack of transportation into the story and consequently limited persuasive power. However, since the videos with the reference to plastic pollution basically represent a local and important issue and the authenticity of the portrayed context and the protagonists in the videos was ensured through cooperation with local stakeholders, a successful immersion can be assumed. Moreover, some of our variables were only measured with a single item. Depending on the specific wording of these items one might find slightly different results (Neef et al., 2023b; Richter et al., 2023c). However, it is unplausible that this would have changed the general outcome of the study.

Furthermore, our study only captured the short-term effects of the intervention. Blythe et al. (2021) indicated that although short-term effects may be observed following a Future Vision intervention, they tend to diminish over the long term, which could hinder the effectiveness of these interventions in promoting long-lasting sustainable behavior change. Long-term effects, even to family members and friends could be captured by adapting a study design with additional measurement points as demonstrated for example by Richter et al. (2023b). Therefore, future research should investigate the potential long-term influences derived from such interventions, for example by performing additional measurements. As demonstrated by Yang et al. (2022), the repeated presentation of environmental videos after a certain duration proved to be a valuable method for enhancing the psychological and emotional involvement of individuals over time. This approach to increase the long-term effects is easy to implement as videos are particularly well suited for multiple distributions and can be easily shared via social media platforms, which would make repeated confrontations with a video intervention feasible. The use of social media campaign to drive behavior change has been proven to be effective (Rapada et al., 2021).

Additionally, further research should explore the effects of optimistic narrative future visions in the context of other sustainable behaviors. This study focused specifically on the sustainable consumption of plastic bags in South Africa, thereby examining a specific context and cultural background. Based on previous work, we are convinced that future visioning is effective especially when it relates to local circumstances and addresses an audience with similar problems in similar environments (Pahl and Bauer, 2013; van Valkengoed et al., 2022). However, further studies investigating other behaviors within the ecological domain could further underline the effectiveness of this approach.

## 6. Conclusion

Plastic pollution in our natural environment is entirely attributed to human activities (see also Pahl et al., 2022). Hence, behavior change is crucial to tackle plastic pollution. How to engage individuals in more sustainable practices through persuasive means of communication is a question addressed in the field of environmental psychology. This study aimed to investigate the impacts of an optimistic narrative future vision conveyed through video media, an intervention recommended by Richter et al. (2023a), to address the escalating issue of plastic pollution in South Africa, specifically focusing on reducing single-use plastic bag consumption. Our research findings contribute valuable insights into the psychological and emotional effects of the Future Vision intervention. Specifically, we found increased positive emotions, PBC and behavioral intentions and decreased negative emotions (we could not find a significant effect for sense of responsibility). These results highlight the approach of using optimistic narrative Future Vision videos as a communication strategy to promote sustainable behaviors regarding disposable plastic bag usage. However, further investigation is warranted to gain a more comprehensive understanding of the effects of video media combined with story-based optimistic future visions on psychological factors and their role in guiding sustainable behaviors. This deeper knowledge would help establish the intervention as an effective tool applicable in various domains such as communication campaigns, political initiatives, and environmental education. Therefore, future research should delve into this promising approach to fully explore its potential and practical implications.

## Data availability statement

The datasets presented in this study can be found in online repositories. The names of the repository/repositories and accession number(s) can be found below: https://osf.io/fsv7z.

## Ethics statement

Ethical approval was not required for the studies involving humans because this is a pure survey study without direct manipulation of the participants. The studies were conducted in accordance with the local legislation and institutional requirements. The participants provided their written informed consent to participate in this study.

## Author contributions

NN and SF wrote the first draft of the manuscript. IR and CR wrote sections of the manuscript. KS provided feedback to the original manuscript. LF was responsible for data analysis. All authors contributed to conception, design of the study, manuscript revision, read, and approved the submitted version.

## Footnotes

1
https://aspredicted.org/648_W6P

2 In South African supermarkets it is common that packers handle the task of packing customers’ purchases.
