# Supplementary material for: Optimistic narrative future visions: a communication tool for promoting sustainable (plastic) behavior
Source: Front Psychol. 2023 Sep 18;14:1252895. doi: 10.3389/fpsyg.2023.1252895 (PMC10543889; doi:10.3389/fpsyg.2023.1252895)

Supplementary Material

Optimistic Narrative Future Visions: A Communication Tool for Promoting Sustainable (Plastic) Behavior

Nicolas E. Neef^1^†, Selina Fußwinkel^2^†, Claudine Roos^3,4^, Lilli Frank^1^, Kapandu Shihepo^3^, Isabell G. Richter*^2^

†These authors contributed equally to this work and share first authorship

^1^ Institute of Education, Work and Society, Dept. of Sustainable Development and Change, University of Hohenheim, Stuttgart, Germany

^2^ Department of Psychology, Norwegian University of Science and Technology, Trondheim, Norway

^3^ School for Geo- and Spatial Sciences, North-West University, Potchefstroom, South Africa

^4^ Unit for Environmental Sciences and Management, North-West University, Potchefstroom, South Africa

*** Correspondence:**Isabell G. Richter
[isabel.richter@ntnu.no](mailto:isabel.richter@ntnu.no)

# Supplementary Tables

**Table 1**

*Participants of the workshop held in October 2022 including affiliations*

| **Workshop Organizers** | | | |
| --- | --- | --- | --- |
| **Name** | **Affiliation** | | **Role** |
| Isabel Richter | Norwegian University of Science and Technology (NTNU), Trondheim, Norway | | Overall and breakout group facilitation |
| Nicolas Neef | University of Hohenheim, Stuttgart, Germany | | Breakout group facilitation |
| Claudine Roos | North-West University (NWU), Potchefstroom, South Africa | | Introduction, breakout group facilitation |
| Kapandu Shihepo | NWU | | Breakout group facilitation |
| Tonje Nerkvern | Student research assistant at NTNU | | Protocol writing |
| Kaya Ott | Student research assistant at University of Hohenheim | | Protocol writing |
| Oliver Riordan | Student research assistant at NWU | | Protocol writing |
| **Invited participants** | | | |
| **Participant No.** | | **Affiliation/ Background** | |
| 1 | Department of Environmental Affairs, Environmental Control Officer  Department of Environmental Affairs  Department of Environment, Forestry and Fisheries, Waste Policy and Information Management  Department of Environment, Forestry and Fisheries, General Waste Minimization  University of Johannesburg, Senior Lecturer  Council for Scientific and Industrial Research, Principal Researcher: Sustainability, Economics and Waste  University of the Western Cape, Professor  Council for Scientific and Industrial Research, Manager Waste RDI Roadmap  University of Johannesburg, Environmental Management  Council for Scientific and Industrial Research. Sustainability, Economics and Waste  Council for Scientific and Industrial Research, Senior Engineer, Life Cycle Assessment  South African Association for Marine Biological Research, Education | | |
| 2 |  |  |  |
| 3 |  |  |  |
| 4 |  |  |  |
| 5 |  |  |  |
| 6 |  |  |  |
| 7 |  |  |  |
| 8 |  |  |  |
| 9 |  |  |  |
| 10 |  |  |  |
| 11 |  |  |  |
| 12 |  |  |  |

**Table 2**

*Results of the Wilcoxon tests (non-parametric test)*

| Study variables | *V* | *p* |
| --- | --- | --- |
| Pos. Emotion | 1552 | < .001 |
| Neg. Emotion | 38652 | < .001 |
| PBC | 4542 | < .001 |
| AR | 4712 | .07 |
| INT | 5039 | < .001 |

# Supplementary Figures

Figure 1

*Histograms displaying the variables distributions*

**
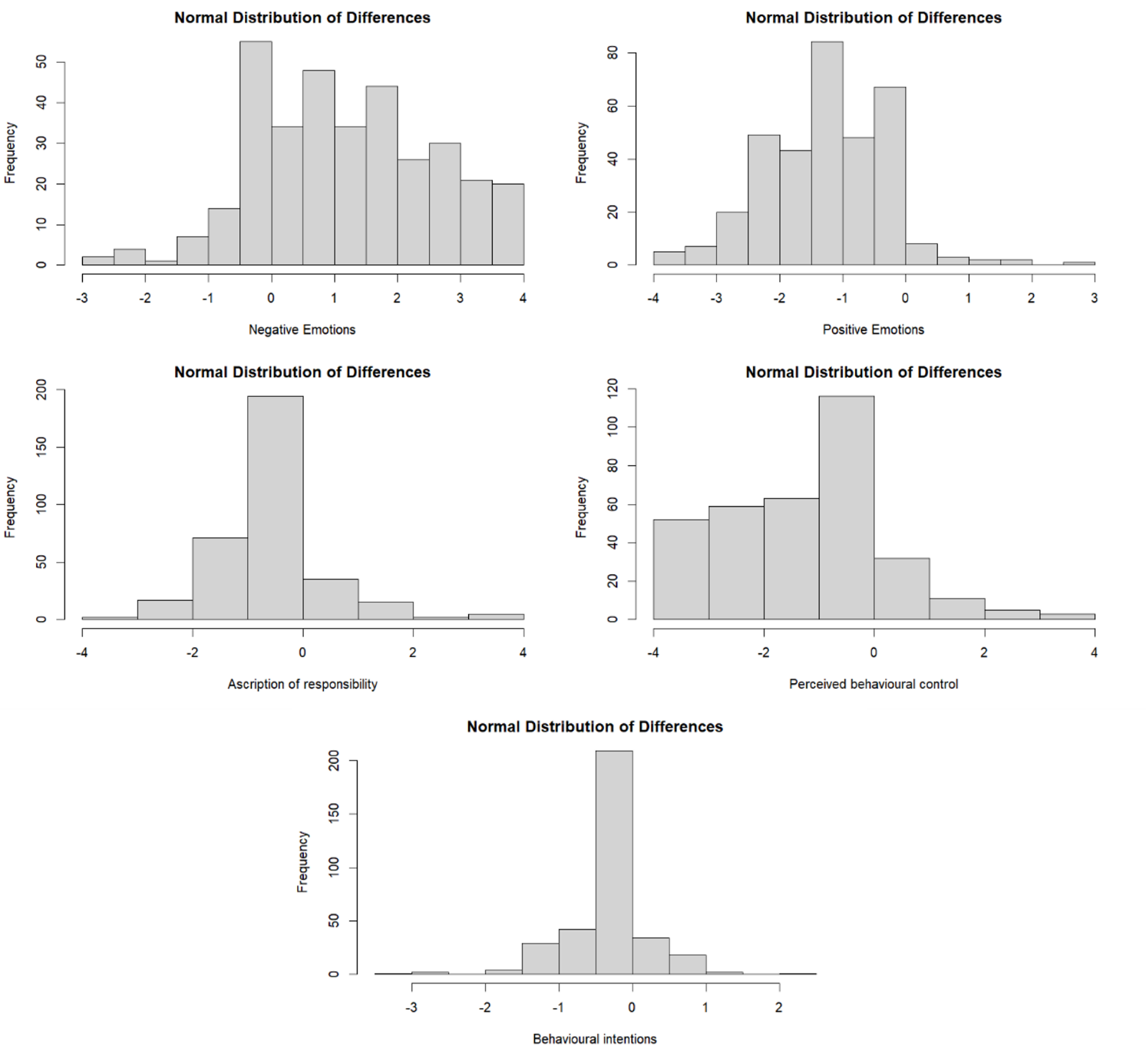
**

Figure 2

*Boxplots before/ after watching the optimistic future vision*

*
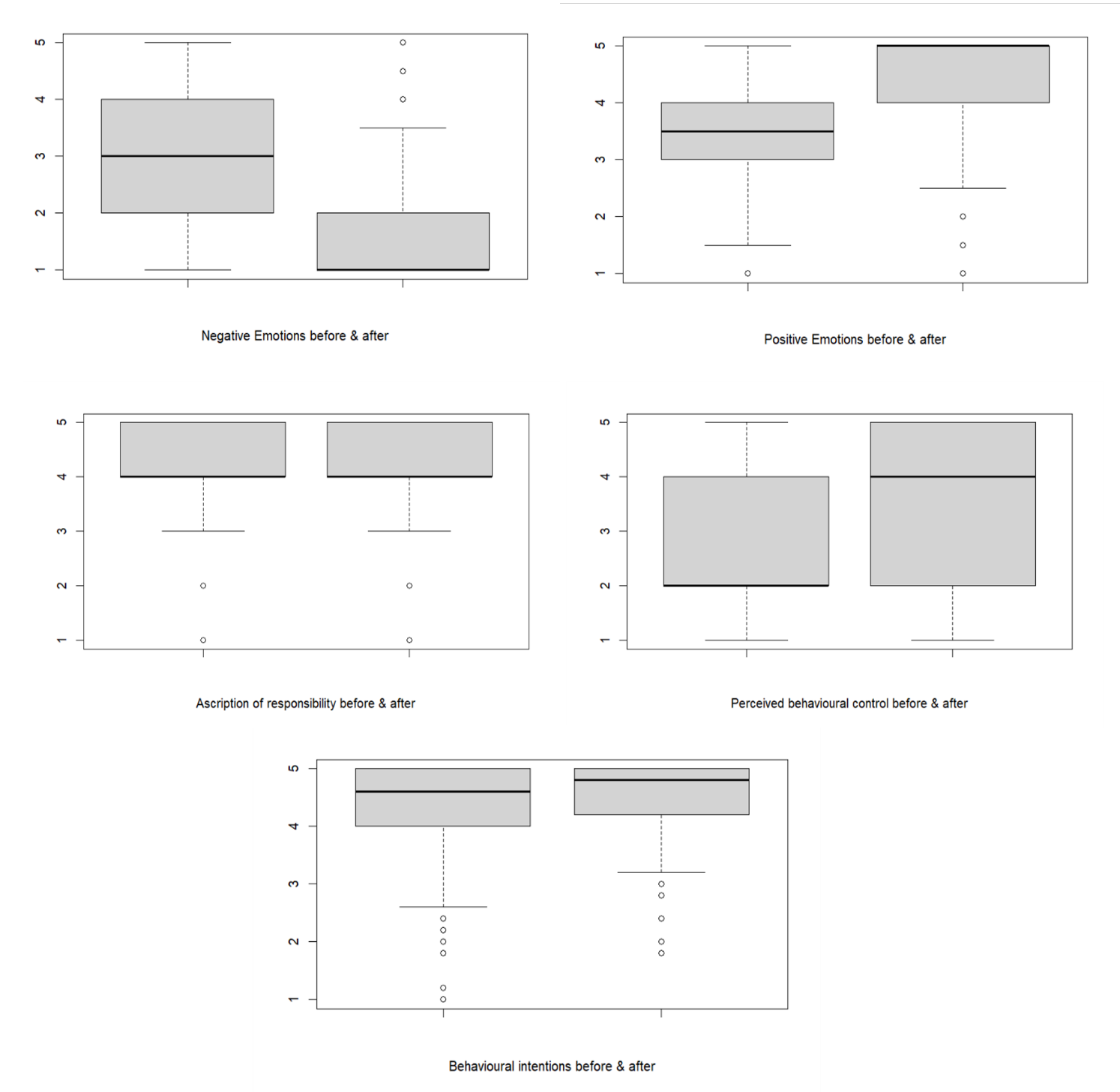
*

# Supplementary Videos

The videos used can be viewed at the following links.

Video 1: <https://osf.io/kug2d>

Video 2: <https://osf.io/g4fe5>

# Questionnaire


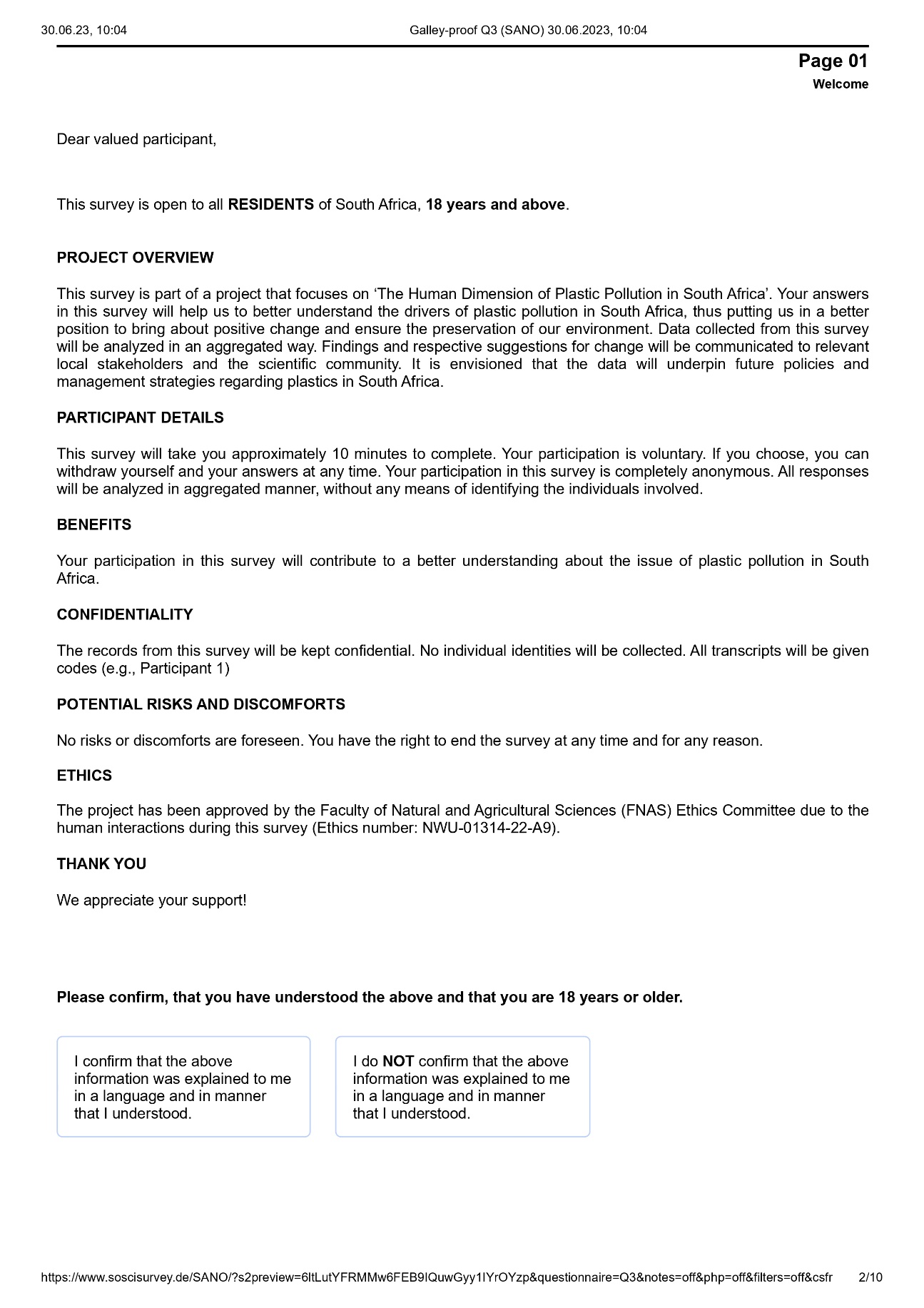


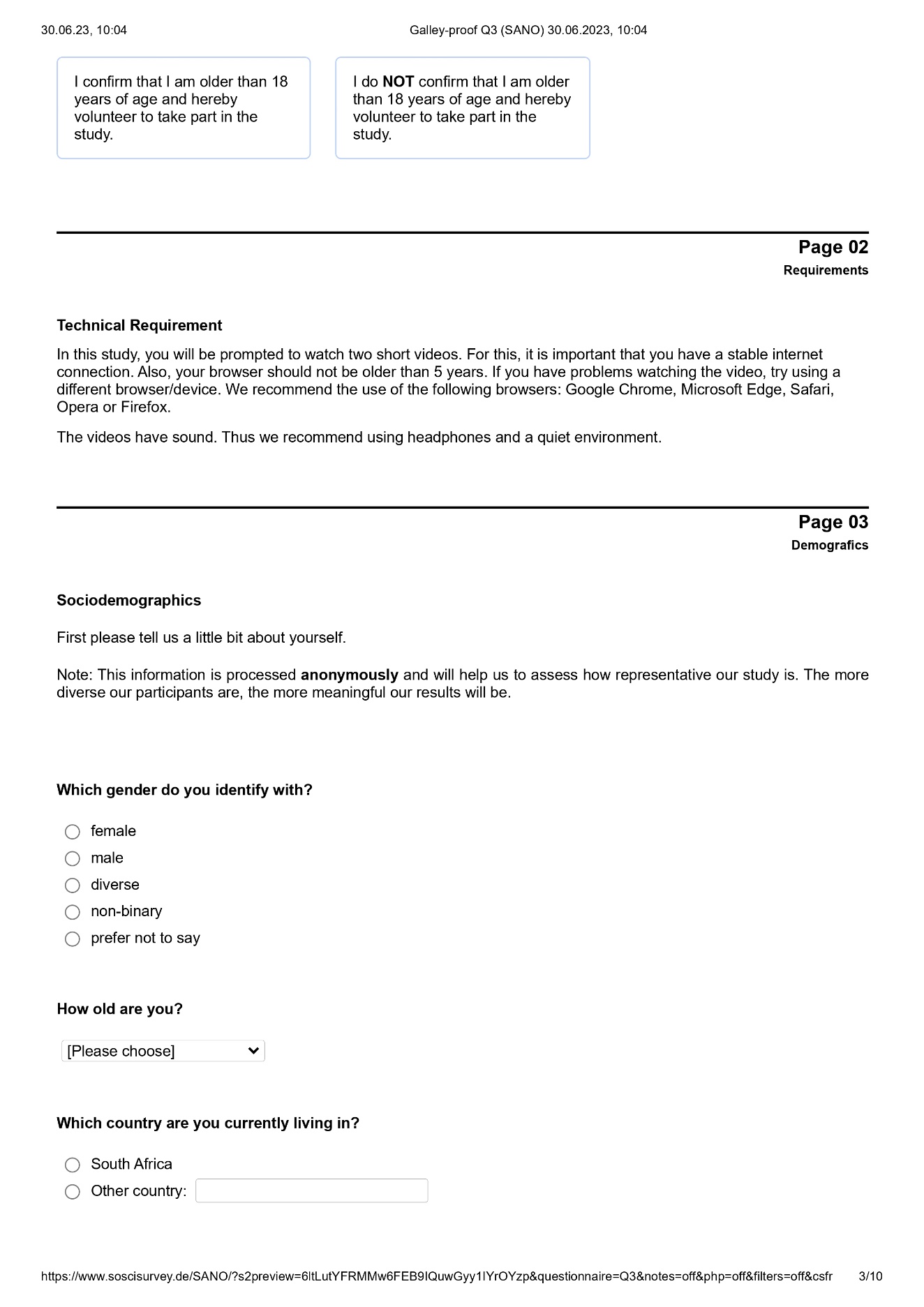

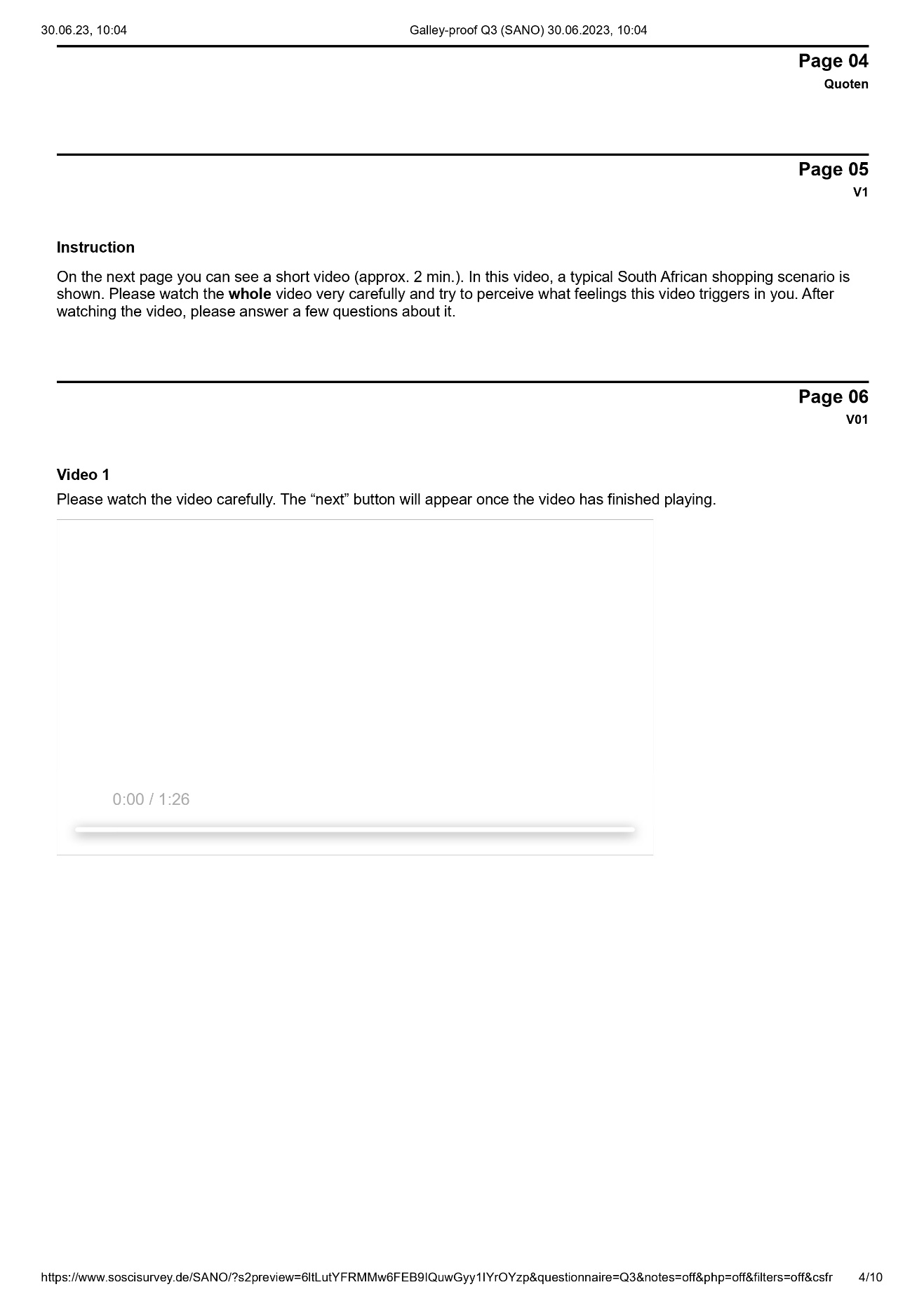

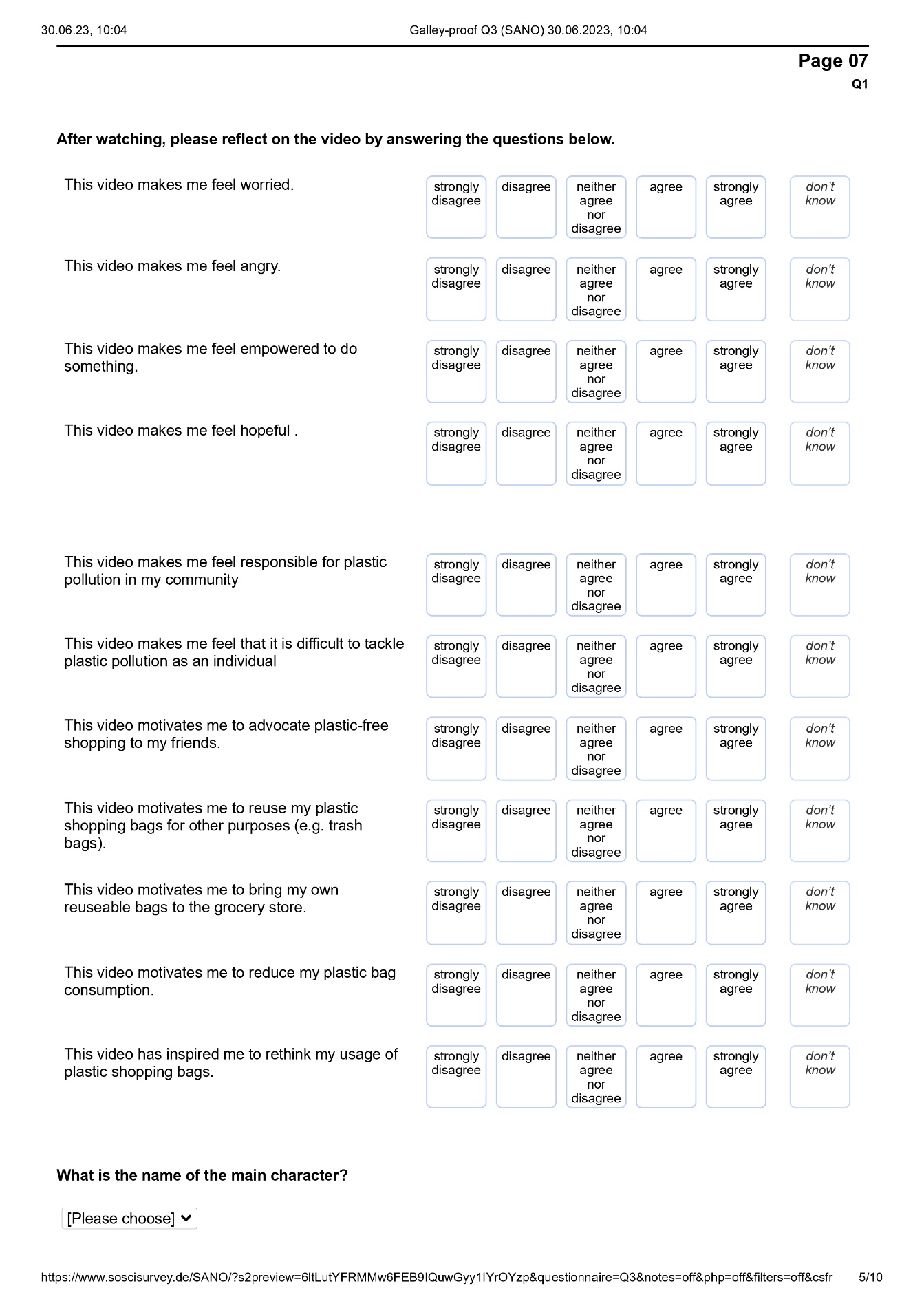

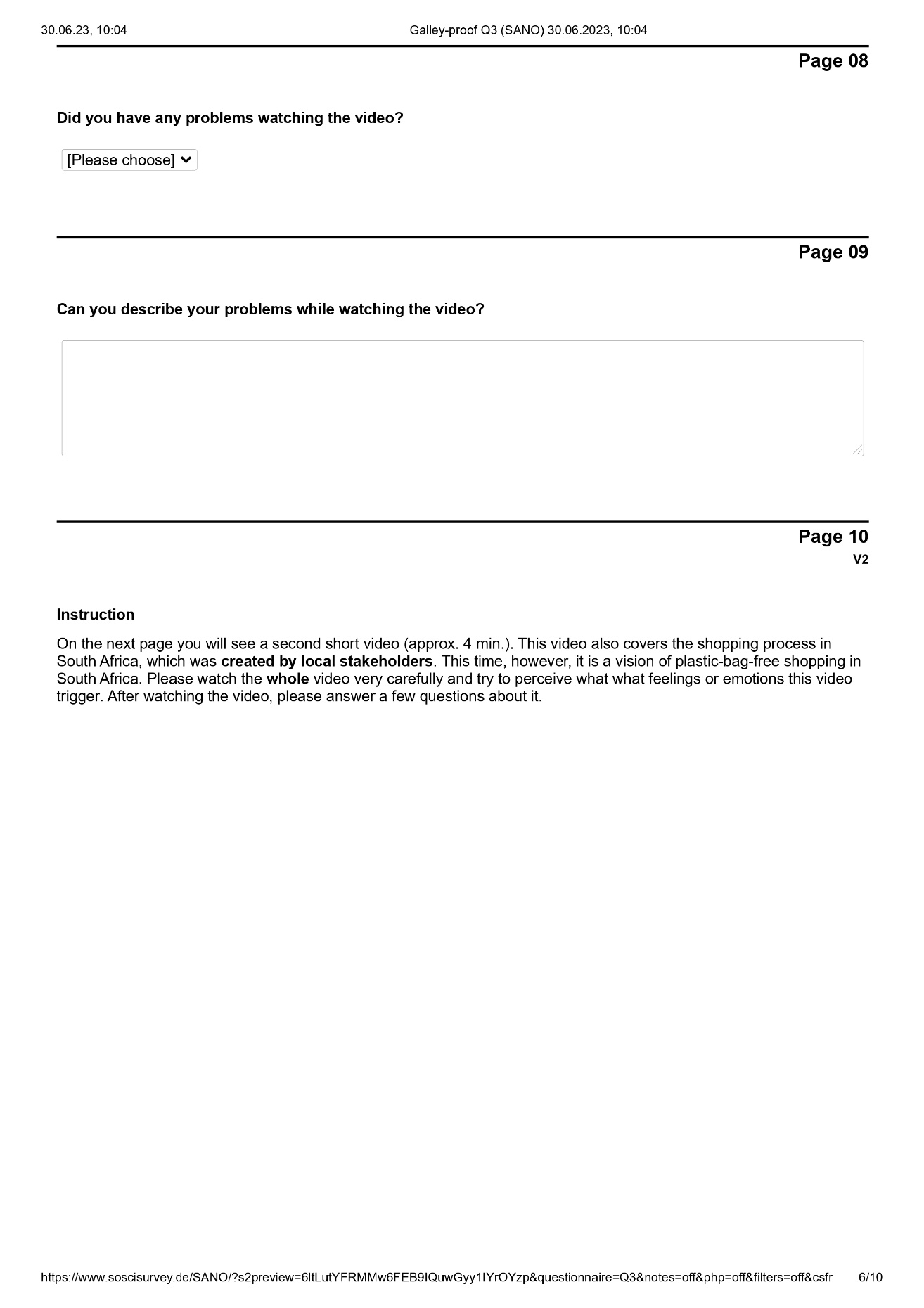

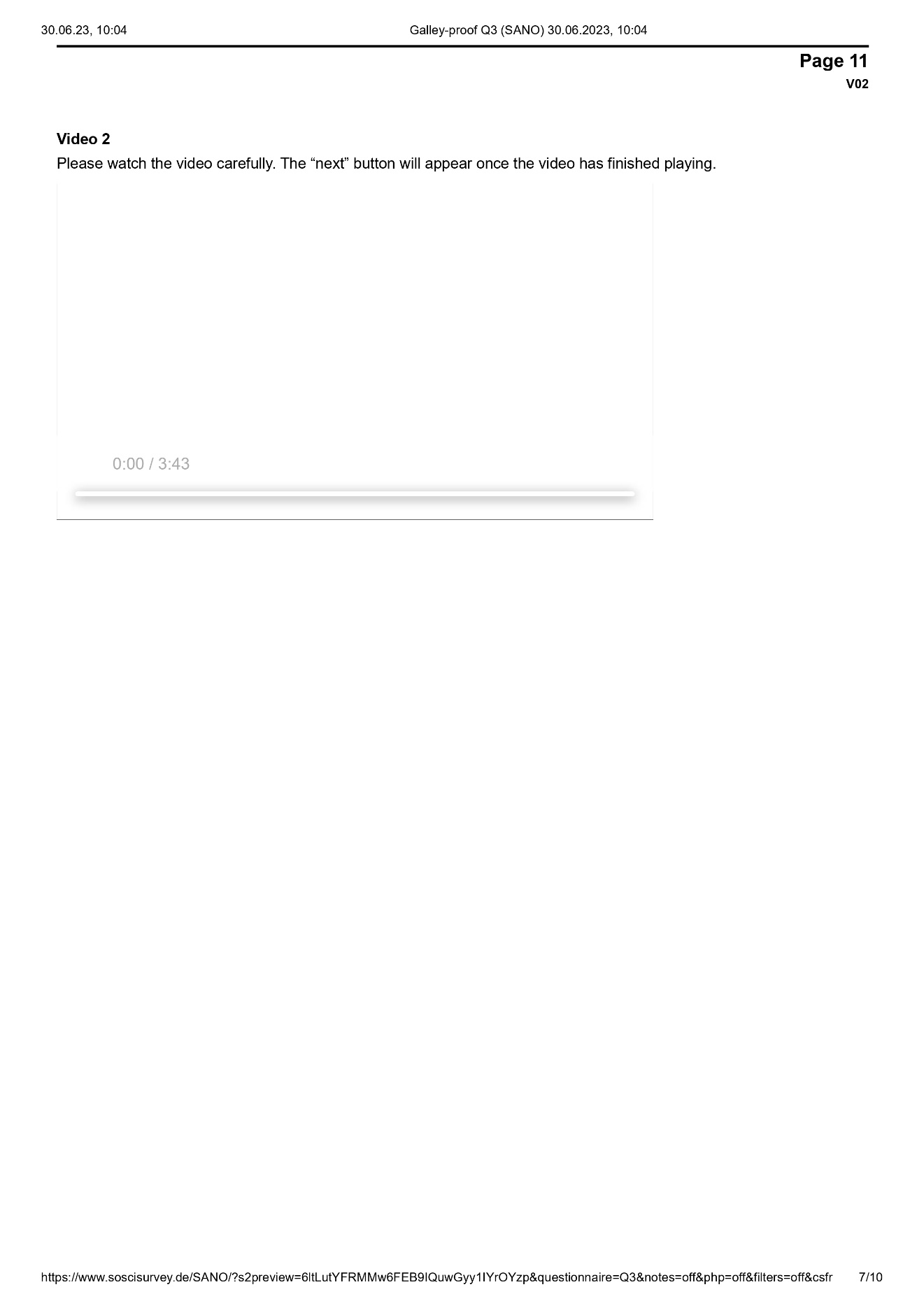

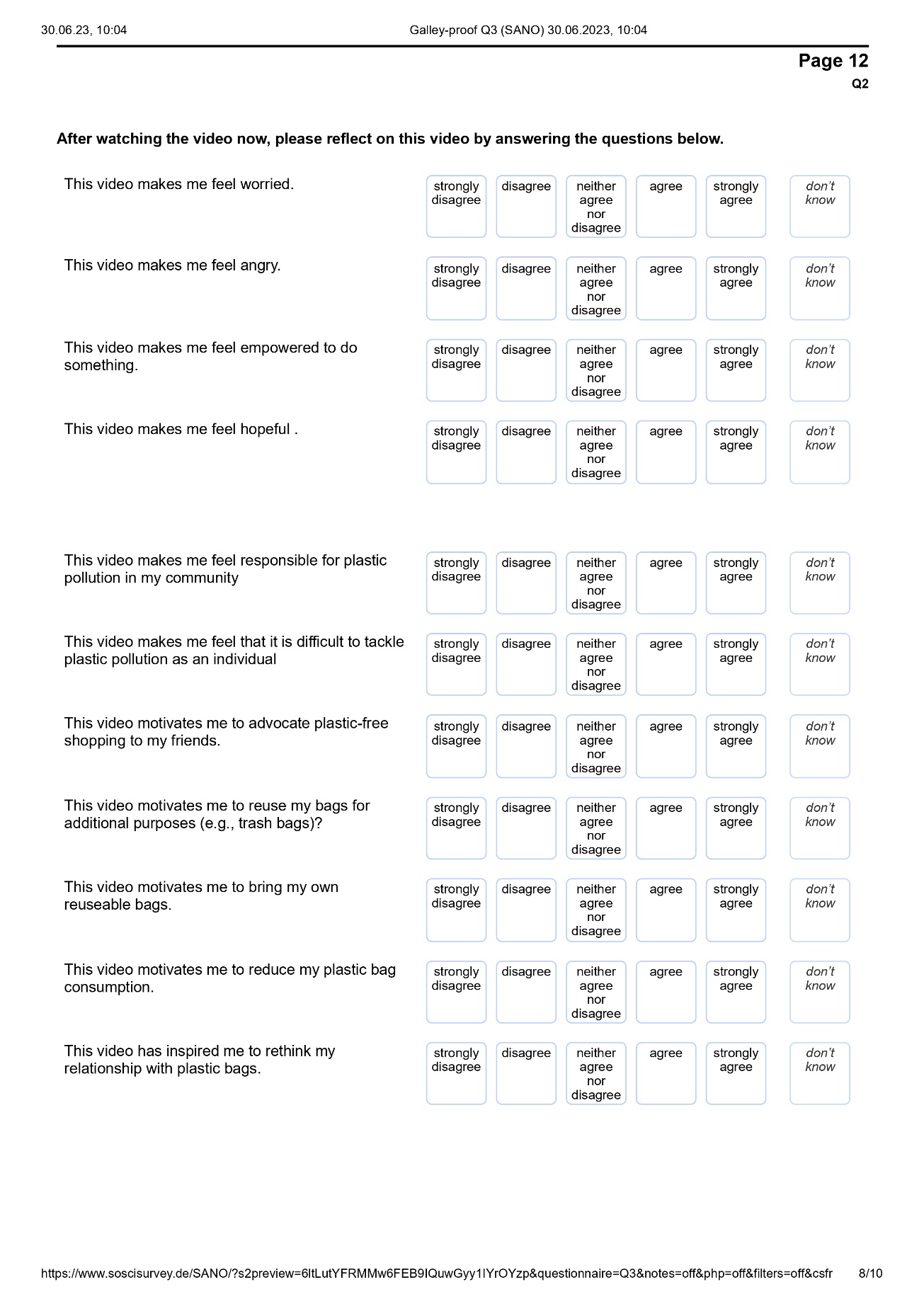

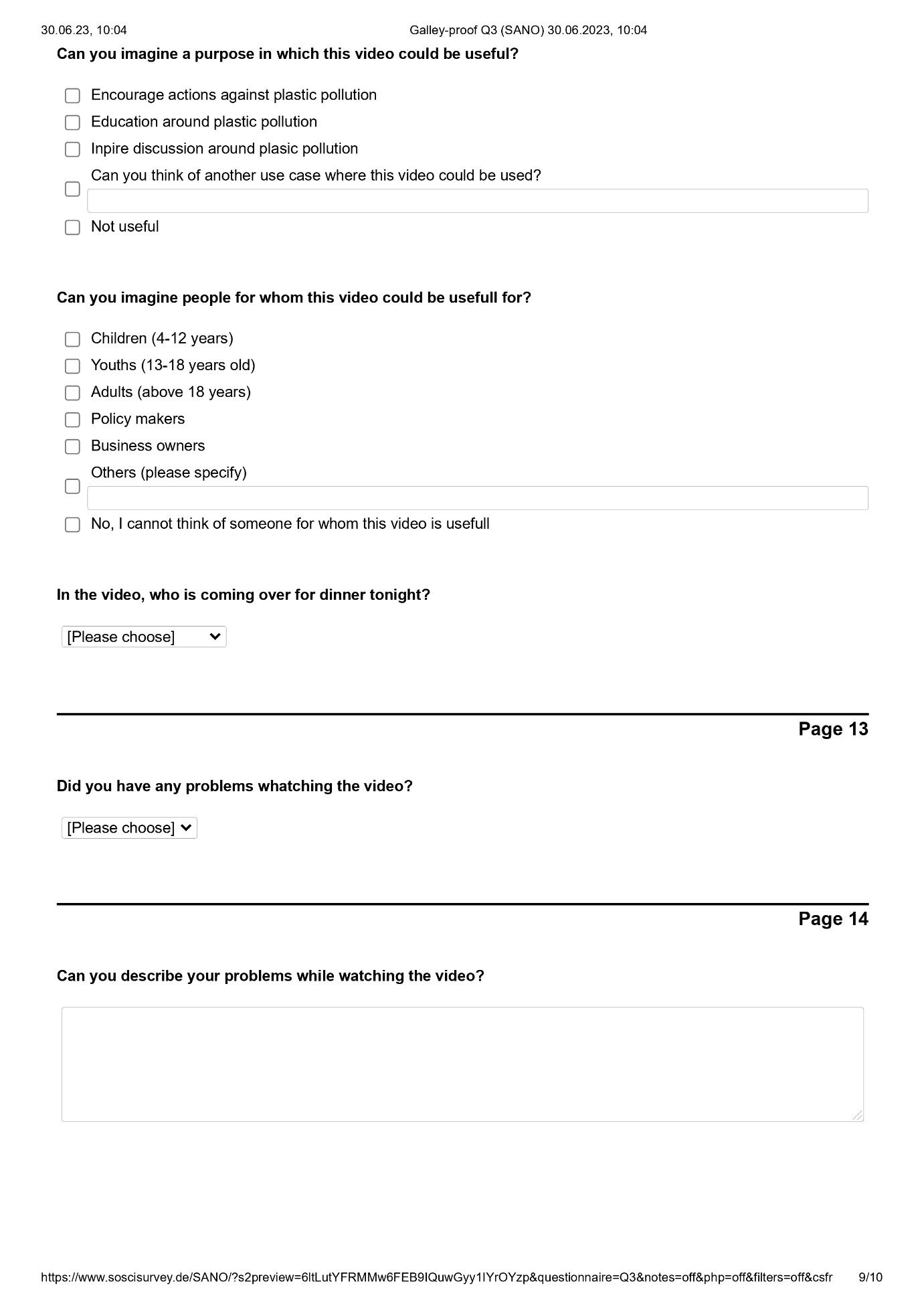

Supplement: Supplementary file 1 [file Data_Sheet_1.docx]
